# Systemic air embolism during percutaneous core needle biopsy of the lung: frequency and risk factors

**DOI:** 10.1186/1471-2466-12-2

**Published:** 2012-02-06

**Authors:** Martin C Freund, Johannes Petersen, Katharina C Goder, Tillmann Bunse, Franz Wiedermann, Bernhard Glodny

**Affiliations:** 1 Department of Radiology, Innsbruck Medical University, Innsbruck, Austria; 2 Department of Anesthesiology, Innsbruck Medical University, Innsbruck, Austria

**Keywords:** Transthoracal biopsy, Computed tomography, Air embolism, Risk analysis, cutting biopsy, Lung Cancer, Smart-Step™

## Abstract

**Background:**

Detection of risk factors for an air embolism in the left atrium, left ventricle, or systemic circulation (systemic air embolism, SAE) during a percutaneous core needle biopsy (PCNB) of the thorax.

**Methods:**

In a retrospective observational study, all PCNBs of the thorax in 610 patients between 2007 and 2009 were analyzed. The SmartStep™ technique (General Electric) was used for the biopsy, with which the examiner can prepare a batch of three 1.25-mm or 2.5-mm collimated slices within a second using a foot switch in the CT room to check the needle position. The image data thus generated and the control CT scans that followed were examined retrospectively for the presence of intravascular air. Intravascular air was defined as two or more adjacent voxels with absorption values of < -200 HU in the left atrium, left ventricle, aorta, or arteries during or after the procedure. The univariate statistical analysis of categorical variables was made using 2 by 2 tables and the Fisher test. The groups were compared using the Mann-Whitney test. Finally, a multivariate logistic regression analysis was used to identify independent risk factors for the occurrence of an SAE.

**Results:**

The radiological incidence of an SAE during a PCNB was 3.8% (23/610 patients), whereas the clinically apparent incidence was 0.49%. Two patients developed clinical symptoms consisting of transient hemiplegia or transient amaurosis; one died due to a fatal SAE of the coronary arteries. The mortality was thus 0.16%. The depth of the needle in the lesion (Wald: 6.859), endotracheal anesthesia (Wald: 5.721), location of the lesion above the level of the left atrium (Wald: 5.159), and prone position of the patients (Wald: 4.317) were independent risk factors for the incidence of an SAE (p < 0.05 each). Using explorative criteria, the acute angle of the needle to the tumor surface, and the transition of ventilated lung were independent factors. The frequency of biopsies, needle penetration depth, and tumor location near the diaphragm or in the lower lobe also played a subordinate role, not however, the needle penetration depth through the lung.

**Conclusion:**

If possible, the PCNB should be performed under local anesthesia. We recommend avoiding endotracheal anesthesia and prone position. Whenever possible, patients should be positioned on the back in such a way that the tumor is lower than the left atrium. The tip of the needle should be within the tumor during the biopsy procedure.

## Background

The occurrence of an air embolism in the left atrium, left ventricle, or systemic circulation during a percutaneous core needle biopsy of the lung (PCNB) [1] with a subsequent systemic air embolism (SAE), with incidences between 0.01% and 0.21%, is a rare, but potentially fatal occurrence [2-8]. The cause of death is cardiac or cerebral infarction [9-11]. Aside from other factors such as accidental intravascular injection and paradox embolism due to a patent foramen ovale [12], three mechanisms in particular are responsible during PCNB: placing the tip of the needle in a pulmonary vein causing air to be aspirated [11,13], the formation of a bronchial-venous [14,15] or alveolar-venous fistula [15] with air passing into the pulmonary vein if the alveolar or bronchial pressure is high or the venous pressure too low [15], and passage of air via the pulmonary capillary bed from the pulmonary artery into the pulmonary venous system [16], e.g. after pulmonary-arterial injection. Risk factors can be biopsy of cystic or cavitary lesions including vasculitic granulomas, coughing during the biopsy, and positive pressure ventilation [17]. However, there is no evidence for any of these factors beyond anecdotal case study, the lowest level according to GRADE [18] or the US Preventive Services Task Force classification. Based on the hypothesis that air embolisms are more frequent than previously assumed [17], in analogy with other centers [17], systematic control CT scans of the entire thorax after PCNBs were introduced at our center in 2007, as has been recommended as standard procedure for this reason for a few months [19]. It was thus possible for the first time to determine the frequency of air embolisms and to search for risk factors.

## Methods

### Patients

Between March 2007 and September 2009, PCNBs were performed at our institution on a total of 610 patients, who were all included in the retrospective study. There were 252 women and 358 men with an average age of 61.7 ± 13.4 years (mean: 63.7; range: 6-87 years). The patients also included children who were all biopsied under endotracheal anesthesia. The study was approved by the local ethics committee.

### Biopsied lesions

Of the 610 biopsied lesions, 553 were lesions not in contact with the pleura or pleural-based lesions, while the remaining lesions were located in the mediastinum. Prior to the biopsy, a bronchial tumor or primary tumor of the lung was suspected in 392 cases (64.3%), metastasis of another malignancy in 108 cases (17.7%), sarcoidosis in 16 cases (2.6%), an infiltrate of unknown origin in 24 cases (3.9%), mycosis in 18 cases (3%), vasculitis in 6 cases (1%), and another disease in 46 cases (7.5%). General anesthesia was usually used to biopsy lesions that were located in the immediate vicinity of the heart, were smaller than 0.8 cm, were located far caudal in the middle lobe or in the lingual, or were allocated to lymph node stations 10-14 in order to avoid major complications from bleeding. Children were also biopsied under general anesthesia. Some patients required artificial ventilation at the time of the biopsy and were already intubated when they came for PCNB.

### Procedure used to prepare for the biopsies

The patients were informed on the day before the examination and gave their written consent. Antiplatelet or anticoagulation drugs such as acetylsalicylic acid, clopidogrel, or phenprocoumon were discontinued at least 10 days prior to the biopsy and generally replaced by low molecular weight heparin. The preconditions for the PCNB were blood count, coagulation, and lung function test. Thrombocytopenic patients were administered platelet concentrates before the biopsy until the platelet count reached at least 60,000/ml. This applied to 7 patients (1.1%). All patients received at least one peripheral venous access for administering contrast medium or fluids and medication in case of complications. For planning purposes, a helical chest CT scan (General Electric LightSpeed 16, Milwaukee, USA) without intravenous contrast administration was performed for every patient in 2.5 mm slice thickness, the results of which were used to make the decision to perform the biopsy. After a positive decision (610 patients; 98.9% of all patients presented for biopsy), we attempted to find a comfortable supine or prone position for the patient. After positioning, which was made on the basis of a planning CT scan, one or two radiopaque bars were taped onto the patient's skin as markers. For the subsequent exact planning of needle pathway an in-plane technique was used and the lesion was scanned once again. These images were searched for a possible needle trajectory that avoided visible bronchi and larger vessels. If a suitable trajectory was not found, the gantry was tilted cranial or caudal as it seemed best for removing the structures to be protected from the biopsy plane, and a new CT scan was made. When a possible biopsy pathway was finally found, at least one other planning CT scan was superimposed on the region in question (helical mode, 2.5 mm slice thickness, fixed tube current of 100 mA) after automatic intravenous injection (Missouri™, Ulrich Medical, Ulm, Germany) of 50 ml Iopamidol 370 (Bracco S.p.A., Milan, Italy) with a flow rate of 2 ml/s for visualizing vital segments of the tumor and to rule out vascular malformations. In the selected slice, the distance between the taped-on marker and the planned needle insertion point was then measured. With the help of the laser indicating the position of the table, this distance was measured on the patients skin using a ruler. This allowed the planned needle insertion point to be marked on the skin. Iodine was applied, the region was covered with sterile drapes, and 2 ml of prilocaine 1% (Xylonest™, AstraZeneca, Vienna, Austria) were applied.

### The SmartStep technique

The biopsy was conducted using the SmartStep™ technique (General Electric, Milwaukee, WI, USA). This makes it possible for the examiner to operate the CT in the examination room with a foot switch. When this switch is activated, three axial slices are displayed on a monitor in the following second. Collimation of 0.625, 1.25, 2.5, or 5 mm can be selected and overlap of 0.625, 1.25, 2.5, or 5 mm can also be selected. Using a manual switch covered with a sterile film, the examiner can pull the patient on the CT table out of the gantry or automatically move him back to his last position. The examiner can freely page through the planning sequence, which can be displayed on the room monitor in parallel. The planning drawing can be displayed and it is possible to window the images. In comparison with fluoroscopy, the advantages of this technique are superior image quality and a lower dose for patient and examiner, but the disadvantage is the delay of up to one second in displaying the images.

### Conducting the biopsies

Using this technique, the coaxial needle (17G, Bard Peripheral Vascular Inc., Tempe, AZ, USA) was usually inserted to the desired position during expiration. Proceeding as described by *Lucidarme et al. *[1], the mandrel of the coaxial needle was then removed. The cannula was immediately closed airtight with a cap (Fresenius Kabi AG, Bad Homburg, Germany) before another control image was made without the mandrel. If the needle was positioned correctly, an initial specimen was taken using an 18G cutting system. The cap was immediately replaced on the coaxial needle and the position was checked. If the needle was not yet in the correct position or if the position had changed, a correction was made. In this way, it was attempted to obtain at least 3 contiguous tissue cylinders. When sufficient material had been removed, 1 ml of fibrin glue was injected per cm of needle track through aerated lung tissue while withdrawing, as described by *Petsas et al. *[20,21] in 1995.

Immediately after removing the needle and applying a sterile adhesive dressing, a control CT scan was made of the entire thorax to detect intravascular air with tube voltage of usually 120 KV and tube currency of 100 mA (0.6 seconds rotation time, 2.5 mm collimation, standard and lung algorithm), and archived in the PACS-system. Depending on the patient's constitution, these parameters were occasionally adjusted if the same image quality could be achieved in slender patients at a lower dose or if a higher dose was required for very obese patients. Two to three hours of bed rest, fasting, and observation were ordered for the patient until a low-dose follow-up CT was made to detect late occurrence of pneumothorax, pleural effusion or parenchymal hemorrhage.

### Special features of the biopsies

If aerated tissue was traversed, fibrin glue (Tisseel™, Baxter Healthcare, Deerfield, IL, USA) was subsequently applied to seal the injection channel, as recommended by *Petsas et al. *[20,21]. For 43 patients (7%), transpulmonary biopsies of the mediastinum were made, mainly of lymph node stations 7 and 10 [22]. Core tissue biopsy needles with a diameter of 17/18G were used together with coaxial biopsy needles with a diameter of 17G (Bard Peripheral Vascular Inc., Tempe, AZ, USA) in 502 cases (82.1%), always in conjunction with a Magnum™ core needle biopsy system (Bard Peripheral Vascular Inc., Tempe, AZ, USA). Indications for endotracheal anesthesia were small tumor size, immediate proximity of the tumor to large vessels of the hilum, mediastinum, or heart, and patient's request or inability to understand the national language. Some patients were already intubated when they came from an intensive care ward. All children were biopsied under endotracheal anesthesia. The PCNBs were performed by four experienced, board-certified radiologists.

### Conducting the examinations and definitions

The examinations were evaluated retrospectively by two observers in consensus on a PACS evaluation workstation (Picture Acquisition and Communication Software; Tiani 3D PACS, Version 3.3.16, Agfa-Gevaert N. V., Mortsel, Belgium) designed for the purpose. Hypodense areas located in the left atrium, left ventricle, or in pulmonary veins which appeared during the biopsy, were visible in at least two consecutive slices, and had Hounsfield levels < -200 HU were considered to be intravascular air. The assessment was verified by a third, very experienced observer (BG). For the diagnosis of intravascular air, all available image data were used; this included images during the SmartStep procedure as well as subsequent thin collimated control CT scans together with coronal, and sagittal reconstructions. All images had been processed in a standard and a lung reconstruction kernel, and all data sets were stored in the PACS system, as well as the original 0.625 mm collimated images. The mean density within the intravascular gas was -383.5 ± 266.2 Hounsfield units. Complications were classified according to the Society of Interventional Radiology Clinical Practice Guidelines of 2003 [23].

### Parameters measured

The parameters explained below were considered as risk factors for the occurrence of an embolism. Under the hypothesis that the probability of injuring a vessel would increase the more biopsies were taken, the number of biopsies taken was recorded. The penetration depth of the cutting needle in the lesion and thus the length of the biopsy cylinder could be varied. It was usually 15 or 22 mm, but for smaller lesions shorter cutting lengths were also used. This parameter was designated the penetration depth of the biopsy needle (mm). The duration of the biopsy is the time between infiltration of the skin with a local anesthetic and removal of the coaxial needle after completing the biopsy in minutes. Size and volume of the tumor were determined at a 3D post-processing workstation (Advantage Windows Version 4.4, General Electric, Milwaukee, WI, USA), and indicated in millimeters or milliliters. Vasculitis, for example Wegener's granulomatosis, and mycosis were considered potential risk factors, as was the presence of necrosis as a categorical variable. Traversing aerated healthy lung tissue versus the direct biopsy of pleural-based lesions without traversing aerated tissue was also considered a risk factor in the sense of a categorical variable. Other categorical variables were prone or supine position of the patient and the differentiation between endotracheal anesthesia or local anesthesia, as well as the completeness of the fibrin line through the lung after removal of the needle. The distance of the tumor from the apex of the lung in relation to the total length of the lung in the body's longitudinal axis was considered, as was the distance of the tumor ventral from the dorsal border of the lung in relation to the total width in the anterior-posterior dimension, and the distance of the tumor mediastinal to the lateral border of the lung in relation to the total area in the coronal dimension. These were relative measures for the location of the tumor in the space of the lung, in the sense of a three-dimensional coordinates system. They were indicated in percentages. For determining these data, strictly transverse, sagittal, or coronal oriented, 3D, minimum-intensity projection reconstructions were made on the aforementioned post-processing workstation. The position of the mid-point of the tumor with respect to the mid-point of the left atrium was taken into consideration. To do this, the distance of the plane parallel to the table top through the center of the tumor from the plane parallel to the table top through the center of the atrium was indicated in millimeters, with a positive algebraic sign if the tumor was closer to the table top than the atrium and a negative algebraic sign if the tumor was higher than the atrium above the table top. In an analogous manner, the vertical distance between the center of the tumor and the tabletop was determined in centimeters. The subjective extent of patient movement on a scale of one to four was recorded. One meant that the patient was very still and did not move; four meant that the patient had moved a great deal during the examination, e.g. by placing his arm, which was positioned over the head, next to his body or vice versa or by changing his position. The pleura-tumor distance in centimeters, the emphysema in the needle trajectory zone through the lung indicated in Hounsfield units, the partial thromboplastin time (in seconds), prothrombin time (%), platelet count, age in years, and sex were taken into consideration. The greatest diameter of any bleeding into the lung was measured in millimeters and considered as a parameter. The smallest angle between the needle and the surface of the tumor at the point the needle entered the tumor was considered as "needle angle to the tumor surface". The slice thickness of the axial images in the SmartStep mode during the biopsy was also recorded. The parameter "coughing" was recorded only rarely; we therefore did not include it in the evaluation.

### Measures in the event an SAE occurred

If an air embolism was detected during or directly after the procedure, immediate measures were initiated. All four cases (4/23) occurred in a prone position. The head was lowered in the Trendelenburg position to prevent a cerebral infarction and the biopsy was terminated. Pure oxygen was administered and intravenous anticoagulation with unfractionated heparin was initiated [24,25]. The position was maintained until all the air was physically reabsorbed. Immediately after this, a neurological examination was ordered. The remaining cases were not detected until the neurological symptoms occurred after the biopsy (n = 2; 2/23), or until the retrospective evaluation of the images.

### Statistics

Descriptive statistics were made using the Excel program (Microsoft, Seattle; WA, USA). All analyses were made for each biopsy. For example, if a re-biopsy was needed for a patient, the re-biopsy was counted as a new biopsy. Using distribution analyses with the aid of D'Agostino-Pearson tests, group comparisons were made using the Mann Whitney U test or Student's t-test [26], where appropriate. The univariate comparison of categorical parameters was always carried out using Fisher's exact test, which was calculated using GraphPad Prism, version 5.00 (Graph Pad Software, San Diego, CA, USA). Logistic regression models were then fitted using standard decision criteria and taking all possibly significant parameters determined by univariate methods into consideration and further selection of parameters was made on the basis of the regression models. Finally, in a forward stepwise selection procedure under consideration of the selected parameters, logistic regression models were fitted to the target variables for "intravascular air". The program SPSS 15 (SPSS Inc., IBM, Chicago, IL, USA) was used for this. P < 0.05 was considered statistically significant.

## Results

### Assigning patients to the two groups

For 23 patients, 8 women and 15 men with a mean age of 56.5 ± 18.1 years, an SAE was verified during or after the biopsy. This is equivalent to 3.8% of all patients. No SAE was detected in 587 patients, 244 women and 343 men with a mean age of 63.7 ± 13.4 years, corresponding to 96.2% of all patients.

### Complications

Hemorrhage was verified by CT scan in 400 patients (65.6%). Of these, 397 cases (99.3%) required no therapy (minor complication A), and 3 required nominal therapy consisting of bronchoscopic suctioning (minor complication B). In one patient, the bleeding had to be treated by microcoil embolization of the intercostal artery. Her hospital stay was extended by more than 48 hours (major complication D).

Pneumothorax developed in 94 patients (15.4%); ten patients (1.6%) had to be treated with a chest tube and 84 patients (89.6%) were observed only. This was classified as minor complication A for 83 patients, as minor complication B for one patient requiring nominal therapy, and major complication D for 10 patients requiring prolonged hospitalization > 48 hours.

### Complications due to intravascular air

**Figure 1:**
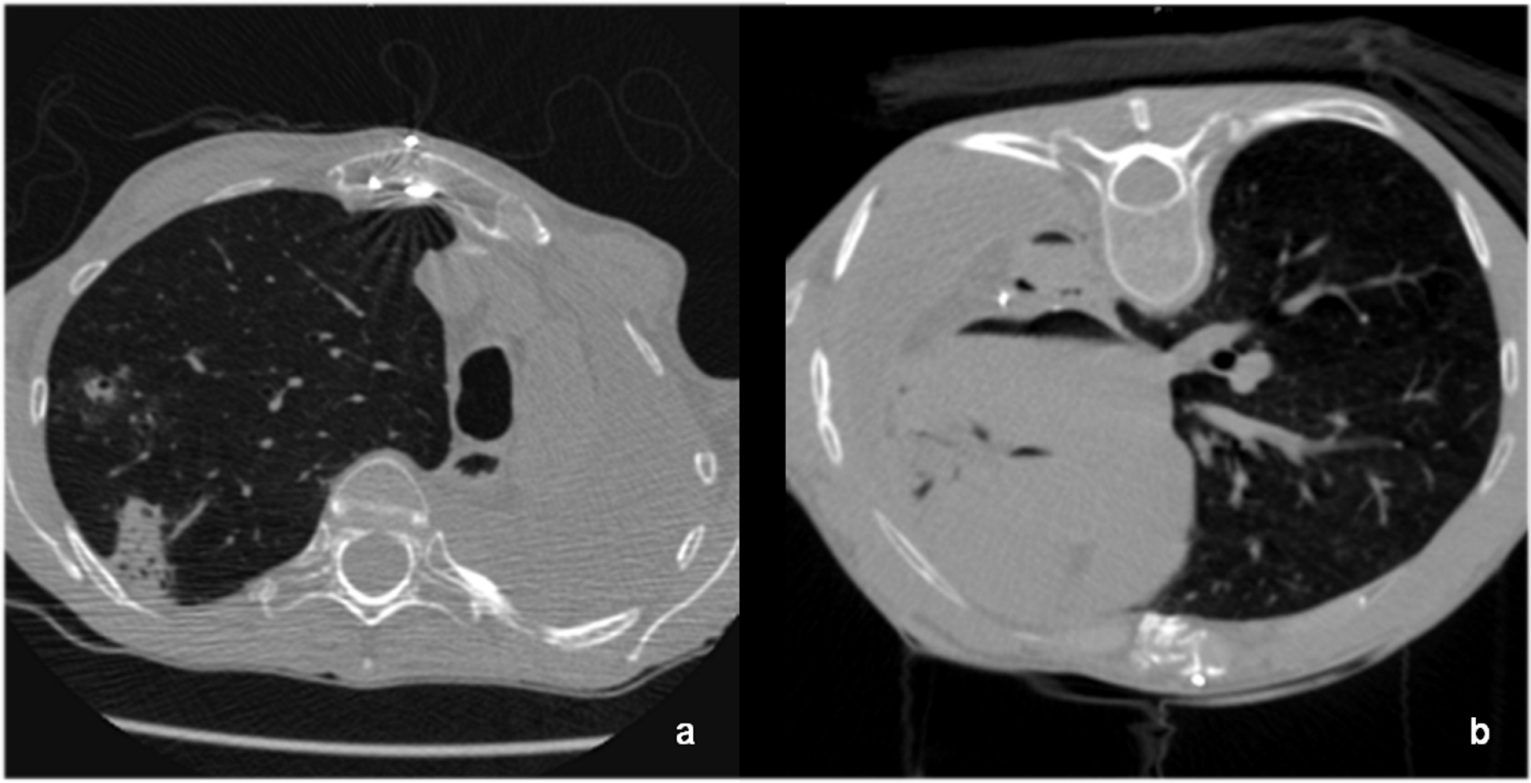
**a and b: Transversal CT-slice, showing SAE in the coronary arteries, the aorta, the left atrium and ventricle (a)**. The lesion was located in the left upper lobe (b), at the time of biopsy approximately 8 cm above the level of the left atrium. 49 y/o patient after lung transplantation, suffering from pulmonary aspergillosis. She died due to coronary ischemia.

While no symptoms occurred in 20 of the 23 patients with SAE, three patients showed symptoms. One male patient developed a transient bilateral amaurosis (major complication D, prolonged hospitalization > 48 hours), one male patient had transient hemiplegia that regressed completely (major complication D, prolonged hospitalization > 48 hours), and one female patient suffered a fatal coronary infarction from an SAE (major complication F, death; Figure 1). The mortality from SAE was thus 1/610 (0.16%), morbidity was 2/610 (0.3%).

### Detection of intravascular air

**Figure 2:**
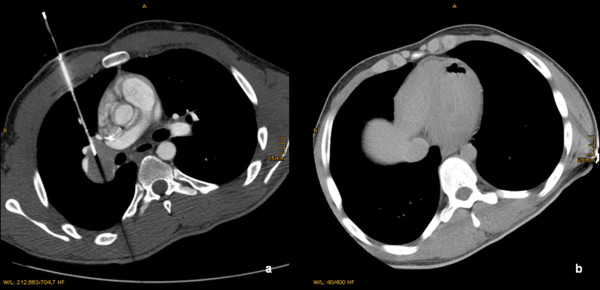
**a and b: Transversal CT-slice of a 29 y/o patient during the biopsy of a tumor located in the right hilum of the lung (a)**. The CT-Scan after biopsy showed a SAE in the apex of the left ventricle (b), which was treated immediately. There were no clinical sequelae.

**Figure 3:**
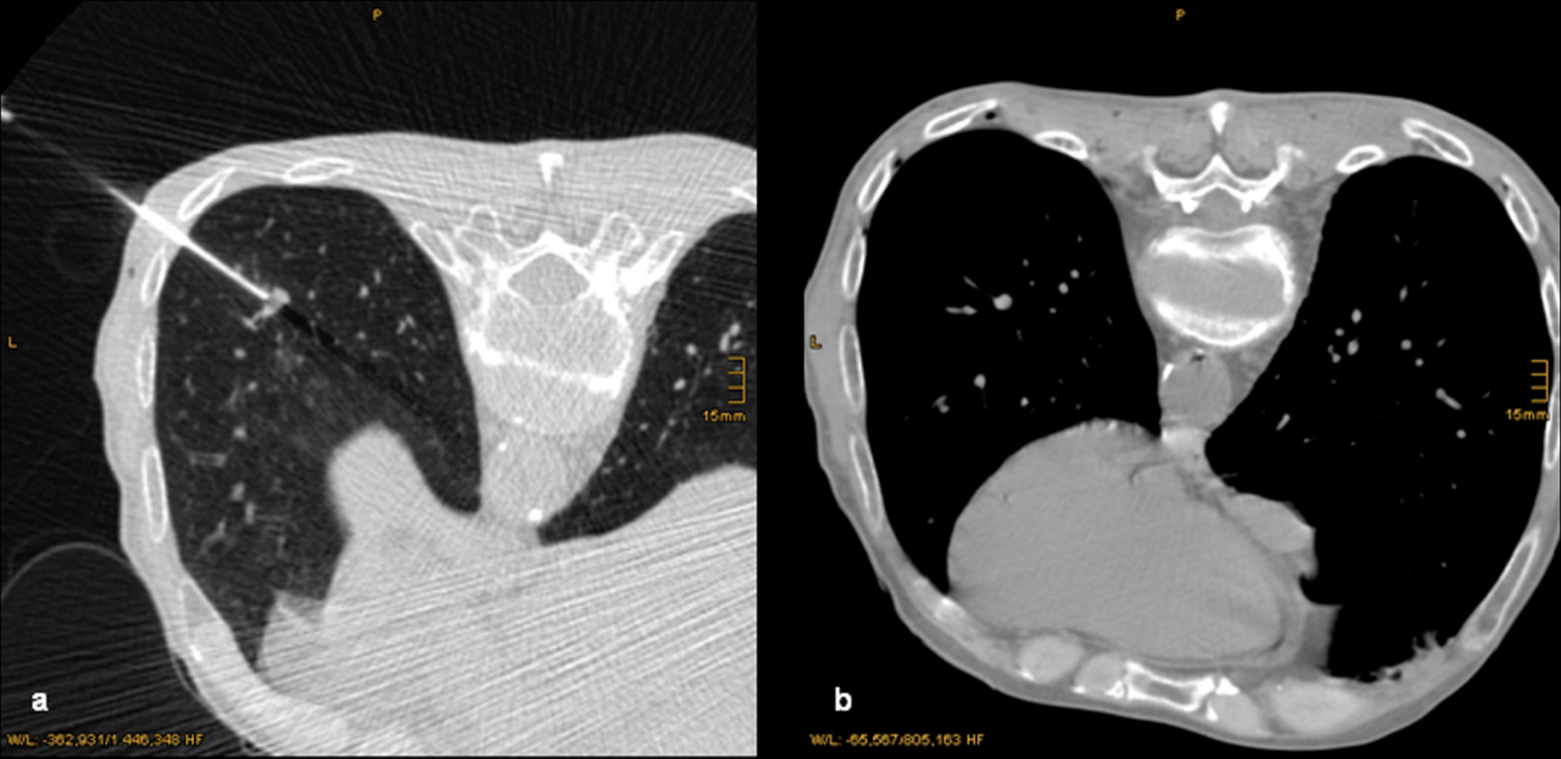
**a and b: Transversal CT-slice of a 48 y/o patient during the biopsy of a small nodule located in the left lower lobe (a)**. The CT-Scan after biopsy showed a SAE in the left atrium, the aorta, and intercostal arteries (b). The patient suffered from a transient amaurosis.

In four patients, the embolism was detected during or directly after the examination on the basis of the CT scans; in two patients, shortly afterward due to the symptoms of amaurosis and cerebral infarction. The other 17 cases were detected retrospectively when evaluating the images in the PACS for this study. Other examples of SAE's are shown in Figure 2 and 3.

### Univariate analysis of the risk factors

In the univariate analyses of categorical variables, prone position of the patient, and location of the lesion in the lower lobe were risk factors for an SAE (p < 0.05 each, Table 1). There was also a tendency seen for the factor endotracheal anesthesia (p < 0.1, Table 1). In the univariate analyses of continuous variables, caudal position of the lesion, level of the lesion above that of the left atrium respective to the patient's position, large number of biopsies, and small CT-slice thickness during the biopsy, were risk factors for an SAE (p < 0.05 each, Table 2). An intravascular position of the tip of the needle was never observed.

**Table 1:** Univariate analysis of categorical data using Fisher's exact test.

	Patients with air embolism			Patients without air embolism			
	Factor present	Factor not present	%	Factor present	Factor not present	%	Significance FET
**Prone position**	19	4	82.6	269	318	45.8	0.0005 (s)
**Lobar location: Upper lobe, middle lobe or lingular segments**	10	13	43.5	391	196	66.6	0.0261 (s)
**Bleeding of any extent visible**	18	5	78.3	382	205	65.01	0.053 (ns)
**Intubation anesthesia**	17	6	74	308	279	52.5	0.0543 (ns)
**Mycosis present**	2	21	8.7	16	571	2.7	0.1443 (ns)
**Necrosis present within the lesion**	6	17	26.0	88	499	15	0.1467 (ns)
**Needle path through ventilated lung**	20	3	87	452	135	77	0.3203 (ns)
**Hemoptysis**	1	22	4.3	11	576	1.8	0.3722 (ns)
**Pneumothorax**	5	18	21.8	89	498	15.1	0.3784 (ns)
**Gender (female)**	8	15	34.8	244	343	41.6	0.6669 (ns)
**Fibrin glue line complete**	17	6	74	404	183	69	0.8185 (ns)
**Vasculitis present**	0	23	0	6	581	1.02	1 (ns)

**Abbreviations: **FET-Fisher's exact test; ns-not significant; s-significant

**Table 2:** Univariate analysis of continuous data using the Mann-Whitney test.

	Patients with air embolism			Patients without air embolism				
	Mean	Median	SD	Mean	Median	SD	Unit of measurement	Significance WMT
**Level of the lesion above the left atrium**	57	63,3	33.2	29.1	33.7	38.9	mm	0.0008 (s)
**Needle angle to the tumor surface**	65.7	68.4	17.2	72.1	75.1	15.7	Angular degree	0.0638 (ns)
**Collimation during the biopsy (smart step)**	2.2	2.5	0.8	2.4	2.5	0.5	mm	0.0318 (s)
**Number of biopsies**	8.9	8	3.0	7.7	7	3.3	number	0.0356 (s)
**Absolute Distance from the apex of the lung to the lesion**	137.4	151	65.5	110.9	104.3	75.2	mm	0.0397 (s)
**Number of specimens**	6.8	7	2.1	6.1	6	2.7	number	0.0569 (ns)
**Relative distance from the apex of the lung to the lesion**	50.6	58.8	22.6	42.6	39.1	24.7	%	0.0606 (ns)
**PT**	108.1	109	13.5	102.9	103	13.0	%	0.1136 (ns)
**Duration of biopsy**	49.3	29.9	60.0	25.9	22	14.5	minutes	0.2461 (ns)
**Patients age**	56.4	62.2	18.0	61.8	63.7	13.4	years	0.2521 (ns)
**Rectangular distance from the plane of the most posterior point of the lung to the tumor (absolute)**	71.6	62.6	41.0	79.5	74.5	41.4	mm	02854 (ns)
**Distance between pleura and tumor in the needle trajectory**	19.8	20.5	13.2	24.5	21.2	19.4	mm	0.3322 (ns)
**Vertical distance between the lesion and the tabletop**	156.5	161	45.2	150.4	145	60.2	mm	03499 (ns)
**Rectangular distance from the plane of the most posterior point of the lung to the tumor (relative)**	37.5	34.2	23.0	40.6	38.2	22.0	%	0.3599 (ns)
**Platelet count**	229.3	234	86.1	254.2	243	88.9	Number/ml	0.4030 (ns)
**Needle deepness in tumor**	10.5	7.1	12.2	7.7	5.3	9.1	mm	0.4589 (ns)
**Degree of movement of the patients**	1.4	1	0.6	1.6	1	0.8	Ordinal scale (1-4)	0.4849 (ns)
**FEV1**	2.4	2.4	0.8	2.3	2.1	0.9	liter	0.4974 (ns)
**Diameter (WHO)**	22.7	18.1	13.1	22.5	17	16.2	mm	0.5597 (ns)
**Density of the lung**	-848.1	-859	43.9	-843.2	-849	50.2	Hounsfield units	0.5602 (ns)
**Axial diameter of the lesion (RECIST)**	32.7	28	23.2	30.0	22.4	23	mm	0.5820 (ns)
**Volume of the lesion**	30.6	6.3	50.9	30.9	5.8	81.9	ml	0.6188 (ns)
**PTT**	29.7	28.5	4.6	29.9	29	4.3	seconds	0.6736 (ns)
**Rectangular distance from the plane of the most lateral point of the lung to the tumor (relative)**	53.1	50.6	17.7	52.1	52.0	19.5	%	0.8185 (ns)
**Diameter of a visible bleeding in lung (RECIST)**	17.5	15.5	13.1	19.4	16.4	20.1	mm	0.8908 (ns)
**Rectangular distance from the plane of the most lateral point of the lung to the tumor (absolute)**	74,9	72	25.9	73.6	73.8	28.1	mm	0.9235 (ns)
**Biopsy length**	16.3	15	3.4	16.7	15	3.3	mm	0.9266 (ns)

**Abbreviations: **FEV1-Forced Expiratory Volume in 1 second; ml-Milliliter; mm-Millimeter; ns-not significant; PT-Prothrombine-Time; PTT-Partial Thromboplasine time; s-significant; WMT-Whitney-Mann Test

### Multivariate analysis of the risk factors

The independent risk factors for the occurrence of an SAE, determined using standard selection criteria in the order of significance, are shown in Table 3. If less restrictive selection criteria were used for including a variable in the model (probability for stepwise entry 0.1 and removal 0.2), the factors needle path through ventilated lung and acute angle of the needle to the surface of the tumor also occur in the models (Table 4)

**Table 3:** Logistic regression model using standard selection criteria with the target parameter "SAE".

	Model	Selection criteria	Wald	p	Assessment
**Needle penetration depth in the tumor (Needle tip not in the tumor is risk factor)**	1	Standard	6.9	0.009	Most important independent risk factor
**Intubation anesthesia (Anesthesia is risk factor)**	1	Standard	5.7	0.017	Independent risk factor
**Height above the level of the left atrium (Height is risk factor)**	1	Standard	5.2	0.023	Independent risk factor
**Prone or supine position (prone position is risk factor)**	1	Standard	4.3	0.038	Independent risk factor

**Table 4:** Logistic regression model using explorative selection criteria with the target parameter "SAE".

	Model	Selection criteria	Wald	p	Assessment
**Needle penetration depth in the tumor (Needle tip not in the tumor is risk factor)**	2	Explorative	9.1	0.002	Most important independent risk factor
**Intubation anesthesia (Anesthesia is risk factor)**	2	Explorative	6.8	0.009	Independent risk factor
**Height above the level of the left atrium (Height is risk factor)**	2	Explorative	6.0	0.014	Independent risk factor
**Needle angle to the tumor surface (More acute angle may be risk factor)**	2	Explorative	4.6	0.032	Independent risk factor
**Prone or supine position (prone position is risk factor)**	2	Explorative	3.3	0.067	Independent risk factor
**Needle path through ventilated lung**	2	Explorative	2.9	0.085	Independent risk factor

## Discussion and Conclusion

This study is the first one to identify risk factors for the occurrence of an SAE that depend on the biopsy technique. Risk factors include needle depth in the tumor, endotracheal anesthesia, level of the tumor above the left atrium, and prone position. In the univariate analyses, prone position of the patient, location of the lesion in the lower lobe, basal location of the tumor, level of the lesion above the level of the left atrium, large number of biopsies were significant. If the presence of intravascular air is investigated, the incidence is clearly higher than previously indicated. However, with a radiological incidence of 3.8%, an air embolism is still considered rare, but its consequences are potentially fatal. In our study, the mortality of 0.16% was attributed to a coronary infarction from an air embolism. However, the morbidity caused by the SAE of one case each of transient amaurosis and transient hemiplegia is comparable with the results of other studies [2-4,4,6-8].

The fact that only 26.1% of the air embolisms were detected during or immediately after the biopsy should draw the conclusion that the radiologist should be more aware of the incidence of SAE during and directly after biopsy and that a meticulous search for intravascular air in the left atrium or left ventricle should be prompted by the radiologist before terminating the procedure. This could prevent the protean manifestations of SAE from the left atrium or left ventricle due to the fact that immediate measures could be initiated while the patient is still on the examination table, for example Trendelenburg positioning, administering pure oxygen and initiating unfractionated heparin i.v. therapy. Probably, these measures might have prevented the neurological sequelae in two study patients with amaurosis and cerebral infarction.

In the systematic retrospective evaluation of the images, the radiological incidence of air embolism was 3.8%, as mentioned above, much higher than the clinically apparent incidence, which was consistent with that indicated in other studies [2-8]. The fact, that many of the SAE's are visible only by radiology in an especially designed protocol must be taken into consideration when the high radiological incidence of air embolisms found here is compared with the clinical data indicated in literature.

Our study represents the first study with a systematic retrospective search for intravascular air embolisms in a study population and with the deduction of risk factors. The knowledge of risk factors allows implementation of adequate measures before the biopsy procedure and may prevent potentially harmful SAE in the future. A good example represents the female patient with SAE to the left coronary artery and subsequent cardiac arrest and death. Three risk factors were present during the biopsy procedure, i.e. prone position, location of the lesion above the level of the left atrium and general anesthesia, but these were not known until this study. Unfortunately, we could not influence any of these factors retrospectively; that is the nature of the retrospective design of the study.

In principle, all the factors named can be easily influenced by the radiologist performing the procedure, however, before drawing any consequences from the study, its limitations should be pointed out. According to the USPSTF classification, the level of evidence is II-2. This is a retrospective observational study, which means that even if it is conducted accurately, due to the non-consideration of factors that, for example, were not documented by the examiner, uncontrolled and uncontrollable errors can arise. Although statistical analyses of the 23 cases with SAE were possible for the first time, it is conceivable that some factors could have been mathematically eliminated from the regression models due to the small size of the patient group with the event. Certain factors of influence such as the biopsy of cavitary or vasculitic lesions [17], whose influence definitely seems plausible, could not even reasonably be accounted for in the models due to their sparsity. On the other hand, it is certainly conceivable that some factors we named might turn out to be non-existent or coincidental in a prospective verification. As can be seen in Table 2 the standard deviation of the factor depth of the needle in the tumor is especially large in the group of patients with an air embolism, and the needle depth in the tumor covered by the standard deviation is largely consistent with the area covered by the standard deviation of the other group - and yet the factor is retained in the multivariate analyses. We have no explanation for this, but we report this result and recommend that it should be confirmed or rejected on the basis of other studies. Another weakness is that no systematic clinical follow-up examinations were made. All patients were seen again by the radiologist in a final consultation two to four hours after the biopsy for a control CT scan, but there are no systematic records of this talk. Unfortunately, there is no record of the probably very important factor "coughing" [27]. Finally, it must be stated that not CT, but transesophageal and probably transcutaneous echocardiography must be considered the gold standard diagnostic measure. Using transesophageal techniques, even the smallest gas bubbles can be detected [15,28,29] and relatively small amounts of air can be detected even transcutaneously [15]. As we did not use these methods, the reported incidence might still underestimate the true occurrence, although storing the thin collimated source images of the control CT scan after the procedure and then having them evaluated retrospectively by three different persons allows us to presume high sensitivity for detecting intravascular air. Finally, we have to consider that among our patients, only 3 showed clinically apparent complications of SAE, too few for further analysis.

The result that endotracheal anesthesia is a risk factor for the occurrence of air embolisms is not surprising, as positive-pressure ventilation has already been suspected as such in literature [17,30,31]. The higher the intra-alveolar or intra-bronchial pressure, the greater the danger of air passing from the aerated areas of the lungs to the vascular system is [15]. Though we have no exact information regarding the ventilation pressure used on our patients, it is advisable to keep it as low as possible. The high percentage of biopsies conducted under endotracheal anesthesia in comparison with literature can be attributed to the large number of complex procedures, such as biopsies of lesions located in close proximity to the heart and tumors that were smaller than 1 cm, and even smaller than 0.8 cm. Additionally, many lesions in lymph node stations 10, 11, 12, 13, and 14 were biopsied, positions in which even the smallest deviations from the plan could lead to severe complications from bleeding. Children, mentally impaired patients, on patient's request or inability to understand the national language were not biopsied under local anesthesia for obvious reasons, nor were already intubated patients from the intensive care ward. In some cases, in the initial medical discussion for a biopsy, patients are advised by their pulmonologist to have "short-acting anesthesia". We have discontinued this liberal handling of endotracheal anesthesia in view of the result that it could be an independent and very plausible risk factor for the occurrence of an SAE. We now advise patients against anesthesia whenever possible and offer analgesic sedation. If endotracheal anesthesia appears to be necessary, the needle is now removed during breath-hold in expiration position after disconnecting the ventilator.

The risk of air inflow to a vein depending on the positioning of the patient is also known from neurosurgery [12]. A sitting position with the head as the highest point of the body is considered especially risky [15]. The frequency of occurrences of intravascular air during PCNB from a tumor located above the level of the left atrium can be explained similarly - the higher the lesion above the left atrium, the lower the pulmonary venous pressure and the greater the danger of air inflow if a vascular wall is damaged. This also explains the risk of the prone position, as most lesions in the lower lobe are biopsied in this position, which are then located far above the level of the atrium. As a consequence, it must be concluded that positioning a lesion above the level of the left atrium and also prone position are to be avoided. Accepting the longer parenchymal path required for the biopsy of dorsal tumors in supine position might thus be safer with respect to the probability of an embolism.

The most important independent risk factor, namely needle penetration depth in the tumor, is very plausible. The deeper the tip of the needle in the tumor was, the lower the risk of an air embolism because no aerated parenchyma was located there and therefore no alveolar-venous or bronchial-venous fistula could be formed. Conversely, when the tip of the needle was outside of the tumor and the biopsy was made from the aerated tissue into the tumor, this was the greatest risk factor. From this it can be concluded that the tip of the needle should be placed within the tumor whenever possible and the aerated lung tissue in front of the tumor should not be included in the biopsy.

The fact that both prone position and level of the tumor above the left atrium are retained in the model independently of one another must be observed more closely. Prone position does not necessarily mean that the tumor was located above the level of the left atrium, and supine position does not necessarily mean that the lesion was located below the level of the atrium, making it plausible that the factors are independent of each other. However, if the factor prone position is omitted from the models, the factor level of the tumor above the left atrium became more important and vice versa. This leads us to conclude that that the factors are not totally independent of each other. Moreover, it is possible that prone position as compared with supine position results in a reduction in compliance of the thorax that changes intrathoracic pressure ratios, so that prone position itself could increase the risk. However, such considerations are highly speculative and are not further supported by our results.

It is theoretically conceivable that by applying fibrin glue during needle removal to prevent pneumothorax, some air could be injected. Arguments against this mechanism are the fact that lack of air was always ensured before the injection and that the injection was administered only while withdrawing the needle to avoid applying a large amount of glue in one place. Since the fibrin glue was not omitted for any patient, the injection with fibrin glue could not be tested in this study with respect to a possible inherent risk. We do not believe that the fibrin glue could have had a significant role because the amount injected was always proportional to the length of the needle track through aerated lung tissue and there was thus covariance with a factor that played no role in the multivariate models. A prospective study of the risk to patients of administering fibrin glue, e.g. by omitting the fibrin glue in randomly selected patients with the advantages reported by Petsas et al. [20,21] in mind appears to be ethically problematic to say the least, and would require a protocol with interim analyses so that the study could be discontinued in the event an increased risk of pneumothorax became apparent.

The use of a coaxial system could be in itself a conceivable reason for the occurrence of an air embolism. The perfect workmanship of these products and the precise fit of the diameter of the cutting needle system to the inner diameter of the coaxial system could lead to the result that when inserting the cutting needle system into the coaxial needle, air is pressed through the coaxial needle into the patient's body. However, in a 10-cm long system, this would be maximum 0.011 cm^3 ^and in a 15-cm long system, maximum 0.017 cm^3 ^- amounts that could not be detected by CT scan even with a slice thickness of 0,625 mm. Furthermore, the number of biopsies, which is a gauge for the procedure of inserting the cutting needle into the coaxial needle, was not a risk factor for SAE. We therefore do not consider the use of a coaxial system to cause air embolisms in lung biopsies.

Subordinate, but conspicuous variables in the univariate analyses that could potentially influence the occurrence of an SAE were the number of biopsies taken and the penetration depth of the cutting needle. This appears to be immediately explainable, as an additional injury is caused with every biopsy or every specimen removed, which can ultimately lead to perforating a pulmonary vein. Increasing the penetration depth of the cutting needle system increases the length of the incision to extract a longer biopsy cylinder and a correspondingly longer section of tissue is injured.

If the tumor is not penetrated in the precise center, which is sometimes necessary because the center of the tumor is necrotic and would not yield any suitable specimens, the vital borders of the tumor are used instead. If the needle is then inserted tangentially into the tumor margin, it is conceivable that due to even a slight deviation, normal lung tissue and the junction from the tumor to the lung would be biopsied. This junction zone contains the vessels that the tumor displaces by its growth, so that an acute angle of the needle to the surface of the tumor also appears to be a plausible risk factor for an SAE, because the closer to the tumor margin the needle is inserted, the more acute this angle is. The position of a lesion near the base of the lung was also a subordinate risk factor. This can be explained by the fact that lung excursions are more pronounced in the basal region than in the apical region, which leads to a greater probability that vessels can be injured by the associated movement.

Since a fine needle biopsy (FNB) was not performed for any of the patients in our study, no statement with respect to the safety of PCNB in comparison to FNB can be made.

Due to the correlations between the position of the lesion in relation to the left atrium, needle angle with respect to the surface of the lesion, frequency of biopsy samples, and penetration depth with the occurrence of air embolisms, we consider the mechanical development of an alveolar-venous fistula to be the most likely reason for SAE [15]. In view of the infrequency of hemoptysis in both groups (Table 1), bronchial-venous fistulas [14,15] appear to be unlikely, as does the pulmonary-arterial injection of air, because we did not find bleeding from the needle track in any patient nor was an intravascular position of a needle tip verified. However it is conceivable that air can enter a pulmonary vein, perhaps invisible due to its small size [11,13], from the tip of the needle or via a temporary alveolar-venous fistula connected with the lumen of the needle. For this reason, the needle must always be closed airtight and should not be left in situ unclosed any longer than absolutely necessary.

One further limitation of the study that should be mentioned is that the results are transferrable only to a limited extent to other centers that use endotracheal anesthesia less often. But because the risk factors level of the lesion above the atrium, positioning the patient in prone or supine position, and needle penetration depth in the tumor were factors independent of this, its significance might even be underestimated in this study out of statistical considerations. Any upward correction of the extent of the influence of these factors must be the subject of new studies, perhaps conducted in parallel in other centers. Conducting a prospective study does not seem to be ethically justifiable in light of our results. Only animal studies with specific individual technical research questions on the topic might possibly be conducted.

In summary, we can state that needle penetration depth in the tumor, endotracheal anesthesia, level of the lesion above the left atrium, and prone position are independent risk factors for the occurrence of an air embolism. Subordinate risk factors could include an acute angle of the needle to the tumor surface, frequency of biopsy samples, penetration depth of the cutting needle, and basal position of the lesion. The radiological incidence of an air embolism is 3.8%, clearly higher than previously estimated, while the incidence of clinically apparent air embolisms was consistent with that reported by other studies. About 15% of patients with confirmed intra-arterial air develop severe and potentially fatal consequences. To prevent air embolisms, endotracheal anesthesia should be avoided whenever possible. We recommend positioning the lesion to be biopsied below the level of the left atrium, and positioning the patient in supine position. The tip of the biopsy needle should be within the lesion during the biopsy procedure and including aerated lung tissue in the biopsy should be avoided.

## Competing interests

The authors declare that they have no competing interests.

## Authors' contributions

The guarantors of integrity of the entire study were MF, BG and JP. They designed the study concept and were the main responsible authors for the intellectual content. They also did the data analysis. All authors were involved in the literature research, clinical research, manuscript preparation and manuscript review. However the main authors editing the manuscript were BG and JP. Data acquisition was performed by KG and TB. The statistical analyses were arranged by MF and BG. All authors read an approved the final manuscript.

## Pre-publication history

The pre-publication history for this paper can be accessed here:

http://www.biomedcentral.com/1471-2466/12/2/prepub
